# Lifestyle and diet

**DOI:** 10.5830/CVJA-2014-063

**Published:** 2014

**Authors:** Lionel H Opie

**Affiliations:** Hatter Institute for Cardiovascular Research in Africa, Groote Schuur Hospital and Medical School, University of Cape Town, Cape Town, South Africa

**Keywords:** diet, cardiovascular risk, Noakes diet, Banting diet, Mediterranean diet

## Abstract

Currently, there is widespread interest in many different diets. The best-known diets include the New Atkins diet in the USA, the Dukan diet in France, and in South Africa the Noakes diet. Two different approaches have emerged, one focusing on a life-long healthy lifestyle and the other emphasising weight loss. These are in fact complementary aims, as will be reviewed and reconciled. Furthermore, besides the dietary approach, there is a valid case for added drug therapy for selected lipid disorders with the use statins. In addition, new drugs are emerging that in the future might eventually considerably reduce the negative health impact of coronary artery disease.

## Lifestyle is life-long

Life-style is life-long in its health implications.^1^ Although diet is only one of the five components of a healthy lifestyle,^2^ diet has recently come to the fore.^3^ When considering overall health, the most important are non-smoking and regular exercise, followed by body weight and diet, in order of importance (Table 1). These proposals are based on a series of important studies on over 100 000 US health professionals over 10 to 25 years, which defined the contribution to health of four major lifestyle factors, only one of which is diet (Table 1).^2^,^4^,^5^

**Table 1:** The ‘big-five’ components of the healthy lifestyle, with contributions of the various components to give protection from risk of death, with and the proposed mechanisms of action. Note that the missing 21% is probably stress related. From Opie,^1^ page 33.

*Lifestyle: ‘big five’*	*Reduced all-cause death risk (%)*	*Mechanism*
Non-smoking	28	Protects arteries
Exercise 30 min or more daily	17	Slows the heart rate, lowers BP
Ideal weight	14	Less toxic chemicals released from fat cells
Ideal diet	13	High unsaturated fatty acids, high vegetables and fruit, low red meat
Modest alcohol	7	Red wine preferred, contains melatonin
All five	79	Remaining 21% may be stress related

While there are many diets to choose from, the majority focusing on weight loss, few diets have had scientifically solid outcome studies to prove that the diet in question actually improves health and increases life span. An exception is the Mediterranean diet, so called because of the very low incidence of heart attacks observed by Ancel Keys in the Mediterranean islands of Corfu and Crete, thus leading to the concept that the Mediterranean diet is an ideal diet,^1^,^6^,^7^ also protecting against heart failure.^8^

## Palaeolithic, the oldest diet

What is the paleolithic diet? Mankind evolved over hundreds of millions of years, therefore the paleolithic diet must have been the standard diet that also evolved over that time. Studies on the teeth of the paleolithic man, as found in East Africa (also in its congener from South Africa), showed that the dental bones and teeth had adapted to process large quantities of low-quality vegetation rather than hard objects.^9^ The paleolithic diet is now recognised as a nutritional pattern based on the ancient diet of wild plants and animals that our ancestors consumed over 10 000 years ago.

In the Kitava dietary study on isolated tribes in Papua, New Guinea, who even recently ate a pre-Westernised diet of 55 to 65% animal foods and 35 to 45% plant foods, these societies had no incidence of stroke, heart disease, diabetes or hypertension.^10^ The diet consisted mainly of fish, grass-fed pasture-raised meats, vegetables, fruits, roots, spices and nuts. There was no restriction on calories or on the foods to be cooked.

Although the Mediterranean diet overlaps with the palaeolithic diet in terms of fibre, antioxidants, saturated fat and mono-unsaturated fat, the paleolithic diet improved glucose tolerance more than did the Mediterranean diet.^10^ Furthermore, this diet is more food satiating than a Mediterranean-like diet in persons with ischaemic heart disease.^11^ Therefore the paleolithic diet both preceded the Mediterranean diet and was apparently better, so it may be that ‘the simpler, the better’.

## Diet and lipids

**Fig. 1.**
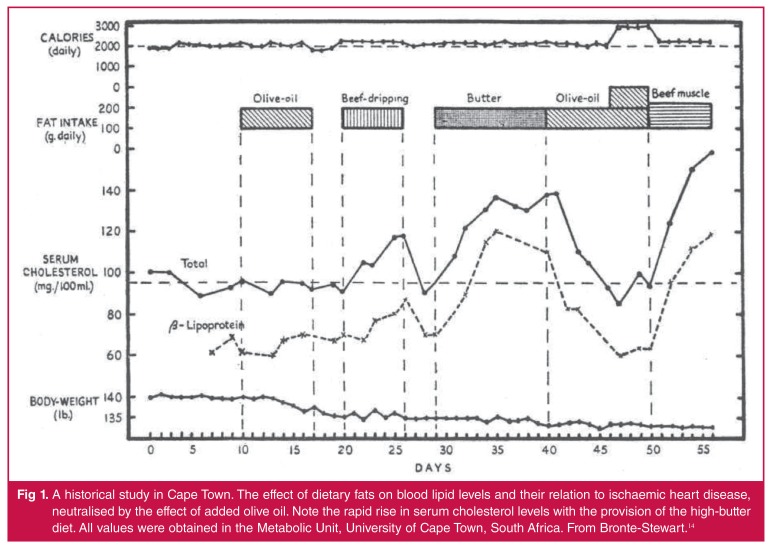
A historical study in Cape Town. The effect of dietary fats on blood lipid levels and their relation to ischaemic heart disease, neutralised by the effect of added olive oil. Note the rapid rise in serum cholesterol levels with the provision of the high-butter diet. All values were obtained in the Metabolic Unit, University of Cape Town, South Africa. From Bronte-Stewart.^14^

Moving on in history, it was the early Cape Town studies that made the link between fat in the diet and blood cholesterol values. Nearly 60 years ago, Professors John Brock and Brian Bronte-Stuart from Groote Schuur and the University of Cape Town Department of Medicine used their specialised metabolic unit to give a high-fat diet to subjects with an initially low blood cholesterol level (Fig. 1).^12^,^13^ A butter load of 100 grams given daily increased blood cholesterol by proximately 40% within five days. The addition of large amounts of olive oil to the butter load restored cholesterol levels to their prior low levels (Fig. 1). Therefore the type of fat diet affected blood cholesterol levels.

The decisive further link between circulating cholesterol values and coronary heart disease came from the Framingham study, which found that higher blood cholesterol values were associated with increased cardiovascular and total mortality rates.^15^ Over time, the emphasis on selection of drug therapy via statins has shifted to the blood level of low-density lipoprotein (LDL) cholesterol.^16^

In South Africa in 2000, high blood cholesterol levels have been estimated to have caused 24 144 deaths (95% CI: 22 404–25 286) or 4.6% of all deaths.^17^ Studies in the Cape Peninsula and in the South African Indian population support links between lipid abnormalities and coronary heart diseases.^18^,^19^ Severely obese South African white women have greatly reduced values for serum high-density lipoprotein (HDL) cholesterol or ‘good’ cholesterol, rather than high levels of LDL cholesterol.^20^

## Lipids in diabetes: the role of statins

The ideal approach to nip diabetes in the bud is by testing HbA_1c_ values in those with the metabolic syndrome or obesity, and then to go for weight loss induced by combined diet and exercise. In those with established type 2 diabetes (DM2), a population study in Hong Kong suggested that statin therapy attenuated the associated increased cancer risk.^21^ For diabetes, in a large study with 215 725 person-years of follow up, statin use before the diagnosis of diabetes reduced diabetic retinopathy (hazard ratio 0.60, 95% CI: 0.54–0.66; *p* < 0.0001), diabetic neuropathy (HR 0.66, 95% CI: 0.57–0.75; *p* < 0.0001), and gangrene of the foot (HR 0.88, 95% CI: 0.80–0.97; *p* = 0.010).^22^ Regarding the general adult population, statins are recommended as first-line therapy in those up to and including 75 years of age, who have clinical atherosclerotic cardiovascular disease (ASCVD) (Table 4 in Stone *et al.*^23^).

## Exercise versus drugs

In studies on the secondary prevention of coronary heart disease and pre-diabetes, randomised trials on exercise interventions suggest that exercise and many drug interventions are often potentially similar in terms of their mortality benefits, rehabilitation after stroke, treatment of heart failure, and prevention of diabetes.^24^ This important observation reinforces the essential role of exercise in any programme aimed at overall cardiovascular health (Table 1).

## Banting first linked diet to mortality

Banting in his pamphlet^25^ in 1869 emphasised the role of diet in weight loss, stating that: ‘The dietary is the principle point in the treatment of corpulence.’ The key points in the Banting diet were his method of reducing obesity by avoiding fat, starch and sugar in the food. Therefore the proposal that the Banting diet is similar to the Noakes high-fat diet3 appears to need re-appraisal. Banting also made wider overall claims that the diet was ‘a simple remedy to reduce and destroy superfluous fat; it may alleviate if not cure gout; prevent or eradicate carbuncles, boils, dyspepsia, makes life more enjoyable, and promotes longevity’. One interesting small but important point is that Banting took the fat off the gravy. For these reasons, it seems preferable to separate the Banting diet from the Noakes low-carbohydrate, high-fat diet.

## Israeli study and new Atkins diet

The low-carbohydrate, high-fat diets that were introduced by Atkins and his successors^26^ have had very wide influence. Some of the key features are as follows, with the relevant book pages given in brackets:

• Protein intake though high has recommended protein ranges (51).
• Fat intake though also high, has a desirable range (70).
• Vegetables including avocadoes are the basis of the permitted carbohydrate intake (102).

In a major landmark Israeli diet, the new Atkins diet was compared with others from the same Israeli population group in a dedicated communal restaurant where the food intake could be monitored.^27^ In the group given the new Atkins diet, besides weight loss, the blood cholesterol pattern showed some favourable changes.

In the comparative group taking a calorie-limited Mediterranean diet, similar changes were found in weight loss and blood lipid levels. However, the Mediterranean diet was calorie limited whereas the Atkins group had a spontaneous loss of appetite. The molecular mechanism to explain the appetite loss is not clear. Reservations are that there was no placebo group and the study was too short to judge any clinical effects on cardiovascular events.

A broadly similar conclusion was reached in a meta-analysis of diets of varying carbohydrate and lipid composition. The new Atkins diet is one of several reduced-calorie diets that have all resulted in clinically meaningful weight loss, regardless of which macronutrients they emphasised.^28^

## What about high-fat weight-losing diets?

The two potential problems with high-fat diets lie in their adverse effects on the blood lipoprotein pattern, and on the impairment of specific mental functions, as observed by Kieran Clarke in Oxford students. In the Oxford study, a short-term, highfat, low-carbohydrate diet led to higher circulating free fatty acid (FFA) concentrations, impaired patterns of myocardial high-energy phosphate metabolism, and decreased cognition in healthy subjects.^29^

The site of these deleterious effects on the brain was the hippocampus. In the heart, sophisticated non-invasive nuclear imaging techniques measured levels of high-energy phosphate compounds, which were relatively low in those taking the high-fat diet. The proposal was that elevated circulating FFA levels were underlying the cognitive and cardiac abnormalities. Therefore Clarke and her associates concluded that high-fat, low-carbohydrate diets are potentially detrimental to the human heart and brain.^29^,^30^

For these reasons, there are arguments to support the view that the diet overweight persons could best start with is a new Akins type of diet for weight loss, coupled with an exercise programme, and then move onto the Mediterranean-type diet to achieve lifelong health benefits, thereby avoiding the cognitive and cardiac changes of high-fat diets. Therefore starting a diet to lose weight, such as the new Atkins or Noakes diet, is complementary with a later switch to the long-term Mediterranean diet. As these diet types come in sequence, they are not competitive.

## The future

A safe prediction is that there will be more editions of existing major books (Atkins in the USA, Dukan in Europe, Noakes in South Africa) besides new diet books. New lipid-lowering pharmaceutical agents are already being tested in large new outcomes-based studies on their preliminary promise.

**Fig. 2.**
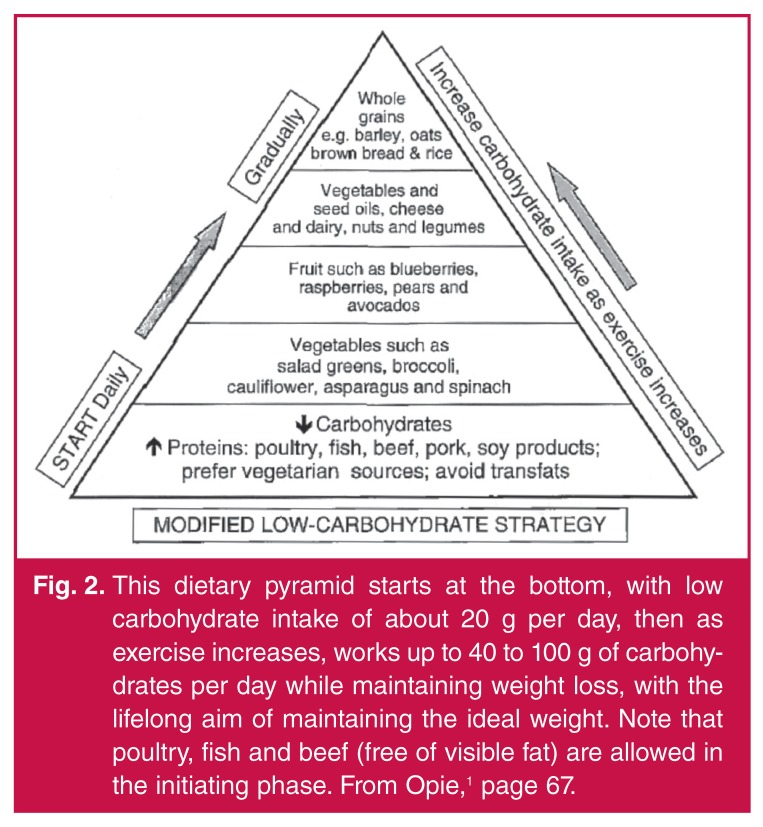
This dietary pyramid starts at the bottom, with low carbohydrate intake of about 20 g per day, then as exercise increases, works up to 40 to 100 g of carbohydrates per day while maintaining weight loss, with the lifelong aim of maintaining the ideal weight. Note that poultry, fish and beef (free of visible fat) are allowed in the initiating phase. From Opie,^1^ page 67.

The best self-help policy may well be to start with a dedicated programme for weight loss however achieved, whether by the new Atkins or Noakes diet, but associated with sufficient exercise. The next step would be to move on to the modified Mediterranean diet (Fig. 2) aimed at living longer and living better.

Looking to the far future, having both fish and meat in the daily diet of large populations would need substantial resources, which will be increasingly limited as the human race expands. Maybe the answer will lie in novel fresh nutritional sources such as algae-based diets.
